# Enhanced supercapacitor performance using EG@COF: a layered porous composite†

**DOI:** 10.1039/d5ra01653c

**Published:** 2025-04-11

**Authors:** Junaid Khan, Anique Ahmed, Abdullah A. Al-Kahtani

**Affiliations:** a Department of Physics Government Postgraduate Collage No. 1 Abbottabad Khyber Pakhtunkhwa Pakistan junaidkhan.nanotech@gmail.com; b Department of Higher Education Achieves and Libraries, Government of Khyber Pakhtunkhwa Pakistan; c Faculty of Engineering Sciences, GIK Institute of Engineering Sciences and Technology Topi 23640 Khyber Pakhtunkhwa Pakistan; d Department of Chemical and Bilogical Engineering, Gachon University 1342 Seongnam-daero Seongnam 13120 Republic of Korea; e Chemistry Department, Collage of Science, King Saud University P. O. Box 2455 Riyadh-22451 Saudi Arabia

## Abstract

In this work, to address the issue of poor conductivity in COFs, a layered porous composite (EG@COF) was successfully synthesized. A redox-active COF (DAAQ-TFP COF) was grown on the surface of expanded graphite (EG) through a solvent-free *in situ* synthesis. SEM analysis displayed that the obtained composite (EG@COF) possessed a layered porous structure. Further investigations revealed that EG not only improved electrical conductivity but also regulated the pore size of the COFs. This structure was highly conducive to enhancing the specific capacitance of the electrode material. An electrochemical study demonstrated that the specific capacitance of EG@COF-3 reached 351 C g^−1^ at 1 A g^−1^, with 94.4% capacitance retention after 10 000 cycles. The excellent capacitance retention was attributed to the stable backbone of the COF. Meanwhile, an asymmetric supercapacitor (ACS) comprising activated carbon (AC) and EG@COF exhibited an energy density of 16.4 W h kg^−1^ at a power density of 806.0 W kg^−1^.

## Abstract 2

In this work, to address the issue of poor conductivity in COFs, a layered porous composite (EG@COF) was successfully synthesized.

## Graphical abstract

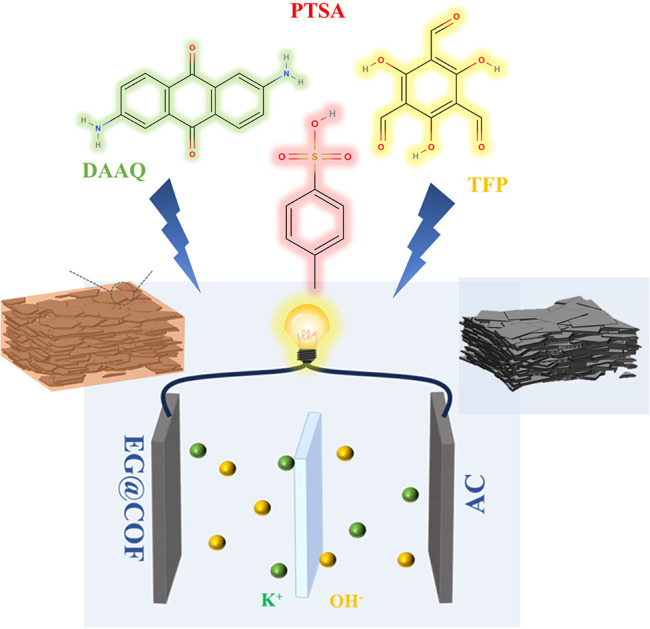

## 1. Introduction

Supercapacitors (SCs) have emerged as a class of highly promising energy storage devices, distinguished by their exceptional rapid charge–discharge capabilities, superior power density compared to conventional batteries (*e.g.*, lead-acid batteries), and remarkable cycling stability, making them well-suited for diverse energy storage applications.^1,2^ However, a critical challenge remains: how to enhance their energy density without compromising their inherent high-power performance.^3–5^ At the heart of this challenge lies the selection of electrode materials, which play a pivotal role in dictating the electrochemical performance of SCs. Thus, the development of advanced electrode materials is of paramount importance.^6,7^

Covalent organic frameworks (COFs) represent a unique class of porous crystalline polymers formed *via* the covalent assembly of organic building blocks.^8^ Since their pioneering discovery by Yaghi in 2005,^9^ COFs have garnered widespread attention due to their exceptional chemical stability, tunable structures, and high specific surface areas.^10^ These advantageous properties have enabled their extensive applications in energy storage, gas separation, catalysis, and beyond.^10,11^ Nevertheless, COFs face considerable challenges in electrochemical energy storage, primarily due to their inherently low electrical conductivity, which hinders their electrochemical performance. Moreover, the availability and utilization of redox-active sites in COFs significantly impact their efficacy as supercapacitor materials.

A notable example is the work by Dichtel's group in 2013,^12^ where they successfully synthesized a two-dimensional COF (DAAQ-TFP COF) with an ultrahigh specific surface area using 1,3,5-trimethylformylresorcinol (TFP) and redox-active 2,6-diaminoanthraquinone (DAAQ) monomers. Despite exhibiting a higher capacitance than non-redox-active COFs, only 2.5% of its redox-active sites were effectively utilized, highlighting the necessity of increasing both the density and utilization efficiency of redox-active sites to enhance electrochemical performance.

In recent years, various strategies have been explored to improve COF-based supercapacitor materials. For instance, Zeng *et al.*^13^ developed a carbon-based material (COF-T800) by carbonizing COFs at high temperatures, which resulted in a 3.3-fold increase in electrical conductivity compared to its pristine COF precursor. Similarly, Sun *et al.*^14^ synthesized COF/GO composites *via in situ* and *ex situ* methods, subsequently carbonizing them to obtain C/rGO materials. Their findings revealed that the *in situ* approach yielded a higher nitrogen content, thereby increasing the density of redox-active sites and significantly improving specific capacitance. Notably, the C/rGO material obtained *via* the *in situ* method exhibited a remarkable capacitance of 234 F g^−1^ at 0.8 A g^−1^, vastly outperforming its *ex situ* counterpart (43.5 F g^−1^). Additionally, the synthesis of COFs, often achieved through Schiff-base condensation reactions, is influenced by several parameters, including reaction time, temperature, and pressure.^15^ Consequently, there remains substantial scope for further exploration and optimization of COF-based materials for supercapacitor applications.^16,17^

Expanded graphite (EG) is a lightweight, high-surface-area material derived from graphite through thermal or chemical expansion, resulting in an interconnected, porous structure with excellent electrical conductivity. When combined with covalent organic frameworks (COFs), the resulting hybrid material benefits from the synergistic combination of EG's conductivity and COFs' highly ordered porosity, structural tunability, and redox-active sites. This enhances ion transport, charge storage capacity, and cycling stability, making the composite an excellent candidate for supercapacitor applications. The presence of COFs introduces additional functional groups that improve electrolyte interaction, while EG provides a robust conductive network, reducing internal resistance and boosting overall electrochemical performance.

In this study, we successfully synthesized an expanded graphite-supported COF (EG@COF) *via* a solvent-free *in situ* approach, facilitating the uniform growth of a redox-active DAAQ-TFP COF on the surface of expanded graphite. The resulting EG@COF exhibited a distinctive layered porous architecture, with scanning electron microscopy (SEM) analyses confirming that its morphology and thickness could be finely tuned by adjusting the proportion of expanded graphite. Optimal conditions enabled the uniform growth of the COF on the EG surface, yielding a well-defined laminated porous structure. The as-prepared EG@COF-3 demonstrated an impressive specific capacitance coupled with outstanding cycling stability. These findings underscore the potential of this synthesis strategy for developing high-performance electrode materials, offering valuable insights into the design of next-generation supercapacitors.

## 2. Experimental

### 2.1. Materials

Expandable graphite (EG), 2,6-diaminoanthraquinone (DAAQ), 2,4,6-triformylphloroglucinol (TFP), and *p*-toluenesulfonic acid (PTSA) were purchased from Shanghai Aladdin Biochemical Technology Co., Ltd. Methanol, acetone, and ethanol were provided by Xilong Chemical Co., Ltd. All reagents were used as received without any further purification.^18^

### 2.2. Pretreatment of expandable graphite

The purchased expandable graphite (1 g) was placed in a crucible and heated in a muffle furnace at 1000 °C for one minute to obtain expanded graphite.

### 2.3. Preparation of COF and EG@COF

Pure COF was prepared based on the report^19^ with modifications. Briefly, DAAQ (0.107 g), TFP (0.063 g), and PTSA (0.5 g) were mixed in 1.0 mL of water. The mixture was then ground for 30 min. The obtained slurry was reacted in an oven at 100 °C for 72 h. The product was washed sequentially with acetone and methanol to obtain pure COF.

**Scheme 1:**
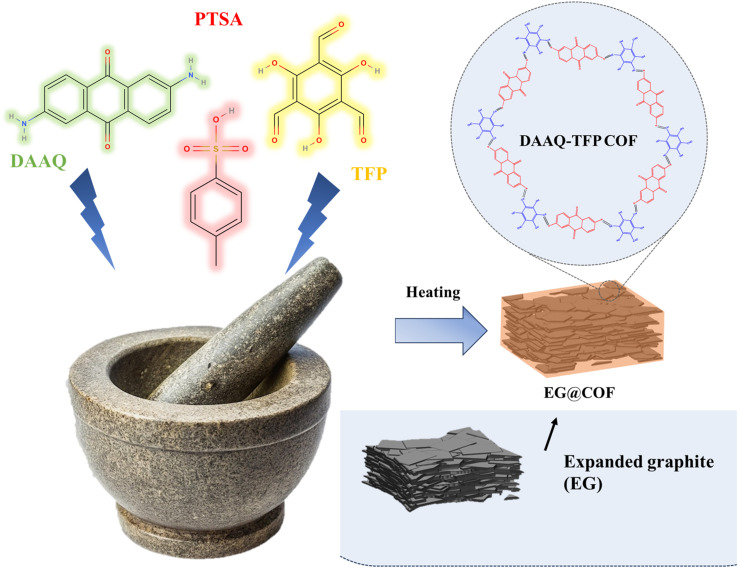
Schematic of the synthesis of EG@COF.

The EG@COF composites were synthesized using a solvent-free *in situ* synthesis method. As shown in Scheme 1, DAAQ (0.107 g) and PTSA (0.5 g) were weighed and added to EG in different amounts (0.0085 g, 0.0119 g, 0.0153 g, 0.0187 g), and the mixture was thoroughly combined in an agate mortar. Subsequently, TFP (0.063 g) was added to the agate mortar, and the mixture was ground for 30 min. The slurry was then heated at 100 °C for 72 h in an oven. The solid obtained was ground and washed with methanol and deionized water to remove PTSA and unreacted reagents. Finally, the powder was vacuum-dried at 60 °C overnight. The prepared composites were labeled as EG@COF-1, EG@COF-2, EG@COF-3 and EG@COF-4, based on the varying amounts of EG used.

### 2.4. Electrochemical measurements

All electrochemical measurements were carried out using a CHI660E workstation (Chenhua, Shanghai) in a 6 M KOH electrolyte solution. The mass-specific capacitance (*C*_g_, F g^−1^) was calculated based on the constant current charge/discharge test data and the following eqn (1):^20^1
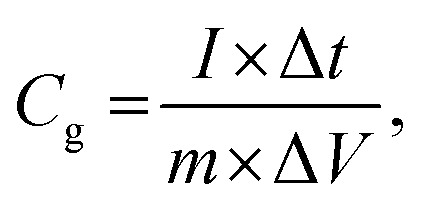
where *I* denotes the discharge current density in amperes (A), Δ*t* denotes the discharge time in seconds (s), *m* represents the mass of the active material in grams (g) (0.004 mg was used in each electrode), and Δ*V* represents the voltage window.^21^

For practical applications, an asymmetric supercapacitor (ACS) was assembled using a two-electrode system. The energy density (*E*, W kg^−1^) and power density (*P*, W h kg^−1^) was calculated using the following eqn (2) and (3):^22,23^2
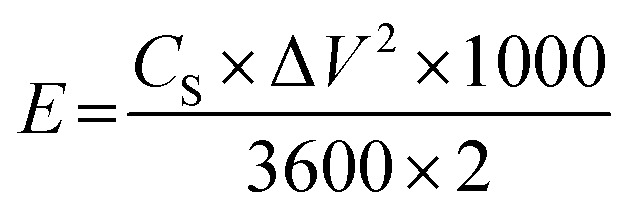
3
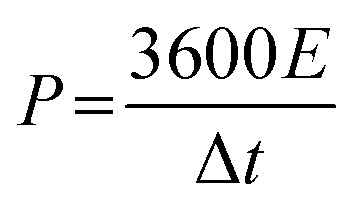


## 3. Results and discussion

**Fig. 1:**
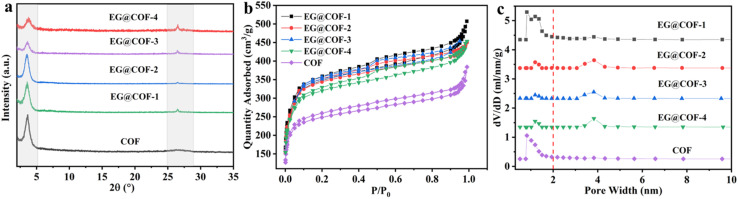
(a) Powder X-ray diffraction patterns of pure COF and EG@COFs, (b) N_2_ adsorption and desorption isotherms recorded at 77 K, (c) pore size distributions of pristine COF and EG@COFs calculated from the adsorption isotherms.

The crystal structures of pure COF and EG@COF were analyzed by powder X-ray diffraction (Fig. 1a). It could be seen that there was a well-defined peak at 2*θ* ≈ 3.6° for all samples, corresponding to the (100) reflection plane of COF, confirming the successful synthesis of COF. For pure COF, there was a broad and weak peak at 2*θ* ≈ 26.6°, attributed to the π–π stacking of the (001) plane of the COF material, which was also consistent with the literature.^24^ For EG@COFs, a distinct characteristic peak appeared at 2*θ* ≈ 26.6° in their XRD curves, resulting from the overlap of the reflection peaks of EG (002) and the COF (001). This characteristic peak became more pronounced as the proportion of EG increased.

The porosity of pure COF and EG@COF was measured by N_2_ adsorption–desorption at 77 K (Fig. 1b). It could be seen that all the adsorption and desorption curves exhibited steep adsorption peaks at low relative pressures (*P*/*P*_0_ < 0.5), characteristic of type I adsorption and desorption isotherms, indicating the presence of micropores in the material. In contrast, the adsorption and desorption curves with the addition of EG showed an obvious hysteresis loop near *P*/*P*_0_ = 0.5, indicating that the material contained mesopores. The pore size distribution of the adsorption–desorption isotherms (Fig. 1c) showed that only micropores were present in the pure COF material, whereas both micropores and mesopores were present in EG@COFs. Combined with the data in Table 1, it could be observed that the addition of EG changed the average pore size of the composites, and the specific surface area increased from 956 m^2^ g^−1^ for pure COF to 1397 m^2^ g^−1^ for EG@COF-3. Among the EG@COFs, the specific surface area of EG@COF-4 decreased, as too much EG was added, preventing the COF from growing uniformly on its surface. Based on the above analysis, EG@COF-3 was selected for further study.

**Table 1:** Summary of specific surface area, average pore size, and coverage ratio of COF and EG@COFs

Material	*S*_BET_ (m^2^ g^−1^)	Pore size (nm)	Coverage ratio (%)
EG@COF-1	1316	0.822	37.7
EG@COF-2	1320	3.829	38.1
EG@COF-3	1397	3.823	46.2
EG@COF-4	1218	3.416	27.4
COF	956	0.757	Reference

**Fig. 2:**
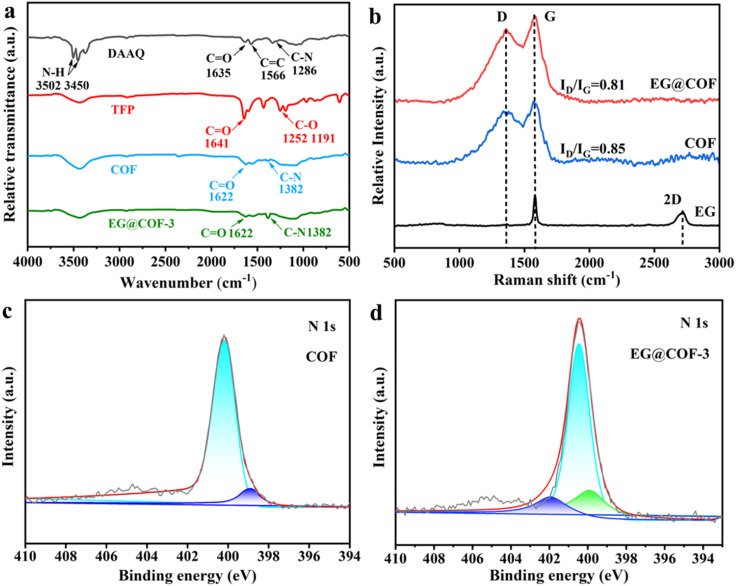
(a) FT-IR spectra of DAAQ, TFP, COF, and EG@COF-3, (b) Raman spectra of EG, pure COF, and EG@COF-3, (c) high-resolution N 1s X-ray photoelectron spectra of pure COF, (d) high-resolution N 1s X-ray photoelectron spectra of EG@COF-3.

The FT-IR spectra of DAAQ, TFP, COF, and EG@COF-3 are shown in Fig. 2a. In the DAAQ curve, the peaks near 3502 and 3450 cm^−1^ indicated symmetric and asymmetric stretching vibrations of –NH_2_. The peaks near to the value of 1635 cm^−1^ correspond to the asymmetric and symmetric stretching vibrations of the C=O bond corresponding to the redox monomer. The C=C stretching vibration of the anthraquinone ring and the C–N stretching vibration of the amino group appeared at 1566 and 1286 cm^−1^, respectively. Furthermore, the disappearance of the N–H absorption peaks (3502 and 3450 cm^−1^) and the generation of a new C–N stretching vibration peak at 1382 cm^−1^ for pure COF and the composite EG@COF indicated that an amidation reaction occurred. Compared to the C=O absorption peaks of DAAQ and TFP (1635 and 1641 cm^−1^, respectively), pure COF and composite EG@COF exhibited a lower energy β-ketoenamine C=O stretching peak at 1622 cm^−1^, indicating the successful synthesis of COF and EG@COF. In addition, the C–O peak in TFP did not appear in the FT-IR spectrum of COF, overall indicating the absence of both DAAQ and TFP monomers.

Fig. 2b shows the Raman spectra of EG, pure COF, and EG@COF-3. In the Raman spectra of carbon materials, the D peak indicates the lattice defects of carbon atoms, and the G peak corresponds to the stretching vibration of sp^2^ hybridized carbon atoms.^25^ The value of *I*_D_/*I*_G_ is typically used to evaluate the degree of defects in carbon materials. In the Raman spectrum of EG, the G peak (2700 cm^−1^) appeared, while the D peak was less prominent, indicating a higher degree of regularity. The *I*_D_/*I*_G_ ratio of EG@COF-3 was 0.81, which was slightly lower than that of COF (0.85). This was due to the large number of oxygen functional groups on the surface of EG, which underwent non-covalent interactions (such as hydrogen bonding, van der Waals forces, *etc.*) with COF. To further understand the bonding state of EG@COF-3, X-ray photoelectron spectroscopy (XPS) analysis was performed. For pure COF (Fig. 2c), the high-resolution XPS spectrum of N 1s could be deconvoluted into two peaks at 399.3 eV and 400.4 eV, corresponding to the N atoms in –NH_2_ and –NH–C, respectively. When EG bound to COF (Fig. 2d), the binding energy of N 1s shifted towards higher energies, with three distinct peaks at 399.8 eV, 400.8 eV, and 401.9 eV. The new peaks that appeared could be attributed to the reaction of the oxygen-containing carboxylic acid groups on the surface of EG with the amine group in DAAQ.^26^ This indicated a change in the bonding pattern of N atoms in EG@COF, proving the chemical connection between EG and COF. This also corresponded to a decrease in the degree of defects in EG@COF, as observed in the Raman spectrum.

**Fig. 3:**
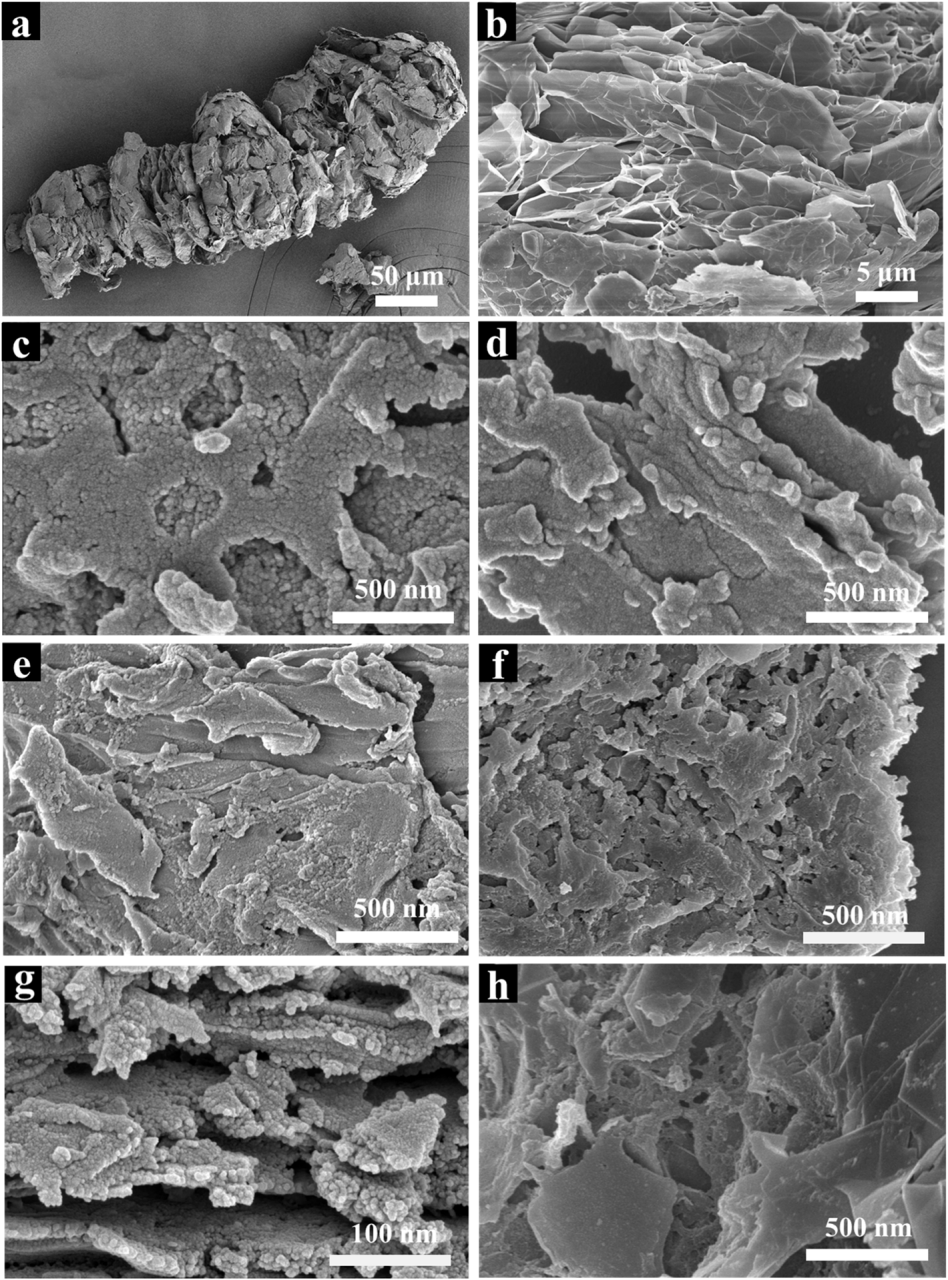
Scanning electron microscopy images of: (a) and (b) expanded graphite, (c) pure COF, (d) EG@COF-1, (e) EG@COF-2, (f) and (g) EG@COF-3, (h) EG@COF-4.

The scanning electron microscopy (SEM) images of expanded graphite before and after milling are shown in Fig. 3a and b, respectively. The morphology of expanded graphite exhibited a worm-like structure, with large pores between the layers (Fig. 3a). After grinding, it transformed into a smooth flake structure (Fig. 3b). The COF material, on the other hand, was granular, with a morphology that showed an aggregated structure formed by the growth of 2D planes and longitudinal stacking (Fig. 3c). The SEM image of EG@COF-1 did not differ significantly from that of pure COF and still showed the stacking morphology of COF (Fig. 3d). In the SEM image of EG@COF-2, it could be seen that this stacking decreased with an increase in EG addition, and the flake structure of expanded graphite, along with the grown COF particles, was observable (Fig. 3e). In the SEM image of EG@COF-3 (Fig. 3f), more holes were observed, indicating that the stacking of COF was reduced and that the COF was able to grow uniformly on the surface of EG. Meanwhile, by observing the side of EG@COF-3 (Fig. 3g), it could be seen that COF particles also grew between the layers of EG. With further increasing the amount of EG, COF could not grow uniformly on the EG surface (Fig. 3h). Based on the SEM image of EG@COF-4, it could be observed that the smoother lamellar structures belonged to EG. The above results implied that an appropriate amount of EG was beneficial for the uniform growth of COF.

It was clear that EG@COF-3 had the optimum morphology. This structure was conducive to increasing the ion exchange rate in the material, which in turn improved the conductivity and electrochemical properties of the material. In addition, the elemental analysis of EG@COF-3 (Fig. S1†) was also carried out, where a uniform distribution of the three elements C, N, and O were observed, further supporting the above analysis.

**Fig. 4:**
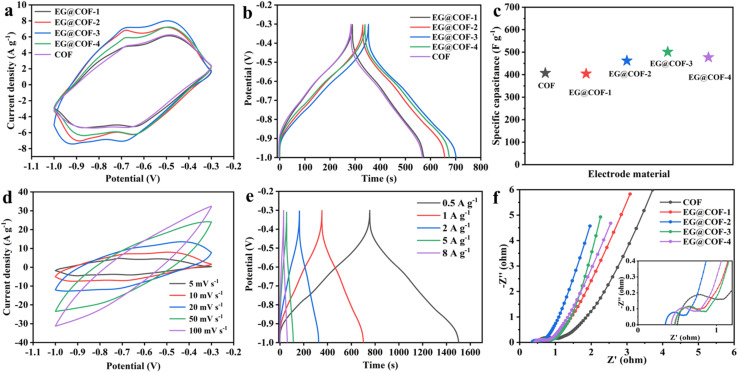
(a) Comparison of cyclic voltammetry (CV) curves of EG@COFs and pure COF at a scan rate of 10 mV s^−1^, (b) GCD curves of EG@COFs and pure COF at different current densities, (c) comparison of specific capacitance values of different materials in this work, (d) CV curves of EG@COF-3 with different scan rates, (e) GCD curves of EG@COF-3 with different current densities, (f) electrochemical impedance spectra of EG@COFs and pure COF.

The electrochemical properties of the samples were tested using a three-electrode system. Fig. 4a shows the CV curves of COF and EG@COFs at 10 mV s^−1^. It could be seen that all of them exhibited two redox peaks. According to ref. 26, it is known that the redox (charge/discharge) mechanism of COF in EG@COF involves the reversible quinone to hydroquinone transformation, as follows (4):4
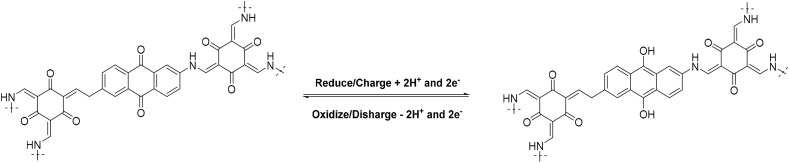


In addition, the curve of EG@COF-3 had the largest peak area, indicating a higher specific capacitance with the largest charge transfer in its electrochemical reaction. Fig. 4b presents their GCD curves at 1 A g^−1^. It was evident that EG@COF-3 had the longest discharge time, and the specific capacitances of COF, EG@COF-1, EG@COF-2, EG@COF-3, and EG@COF-4 were 407, 404, 462, 501 and 477 F g^−1^, respectively (Fig. 4c). The specific capacitance was recorded as 285, 283, 351 and 334 C g^−1^ respectively. The CV curves of EG@COF-3 at different scanning rates are summarized in Fig. 4d. These CV curves showed no obvious changes at scanning rates of 5–50 mV s^−1^, indicating that EG@COF-3 had good electrochemical reversibility. However, the CV of EG@COF-3 showed signs of polarization only when the scanning rate reached 100 mV s^−1^. According to the GCD plots of EG@COF-3 (Fig. 4e), they did not show distortion with the increase in current density (even at 8 A g^−1^). Moreover, the specific capacity was 375, 351, 329, 295, and 288 C g^−1^ at 0.5, 1, 2, 5, and 8 A g^−1^, respectively.

In addition, the capacitance of EG@COF-3 prepared in this study exceeded the capacitance values previously reported in the literature (Table 2). Furthermore, the specific capacitance of EG@COF-3 retained 82% of the original specific capacitance in the range of 0.5–8 A g^−1^. The excellent specific capacitance and retention rate were attributed to two main factors: first, the expanded graphite provided a good growth substrate, altering the pore size of the COF and improving the ionic conductivity of the composites; second, the increase in the specific surface area promoted the exposure of the active sites and facilitated the faradaic reaction. Subsequently, the kinetics of EG@COFs were further investigated using the equivalent circuit model and electrochemical impedance spectroscopy (EIS) spectra (Fig. 4f). According to Fig. 4f, EG@COF-3 exhibited the lowest charge transfer resistance (*R*_ct_, 0.15 Ω). In contrast, the *R*_ct_ of EG@COF-1, EG@COF-2, EG@COF-4, and COF were slightly higher (0.20 Ω, 0.25 Ω, 0.19 Ω, and 0.33 Ω, respectively). As shown in the low-frequency region, it was clear that EG@COF-3 had a steeper slope, while COF showed the smoothest slope. This proved that EG@COF-3 had the lowest diffusion resistance. The addition of expanded graphite enhanced the conductivity of EG@COF, and the amount added also influenced the magnitude of the diffusion resistance. Additionally, a further cycling stability test of EG@COF-3 was carried out (Fig. S2†). The result showed that EG@COF-3 had a 94.4% capacitance retention after 10 000 cycles.

**Table 2:** Comparison of the electrochemical energy storage performance of COF-based electrodes in a three-electrode system

Material	Electrolyte	Potential window (V)	Current density (A g^−1^)	Specific capacitance (F g^−1^)	Ref.
DAAQ-TFP COF	1 M H_2_SO_4_	−0.3–0.3	0.1	48 ± 10	12
N-Doped C/rGO	6 M KOH	0.2–0.6	0.8	234	14
cCNT@COF	0.5 M H_2_SO_4_	−0.3–0.3	0.5	376	27
DAAQ-COFs/GA	1 M H_2_SO_4_	−0.5–0.5	1	378	27
PDC-MA-COF	6 M KOH	0–0.5	1	335	28
COF_BTA-DPPD_–rGO	2 M KOH	0–0.5	0.5	239.1	29
EG@COF	6 M KOH	−1–0.3	1	501	This work

**Fig. 5:**
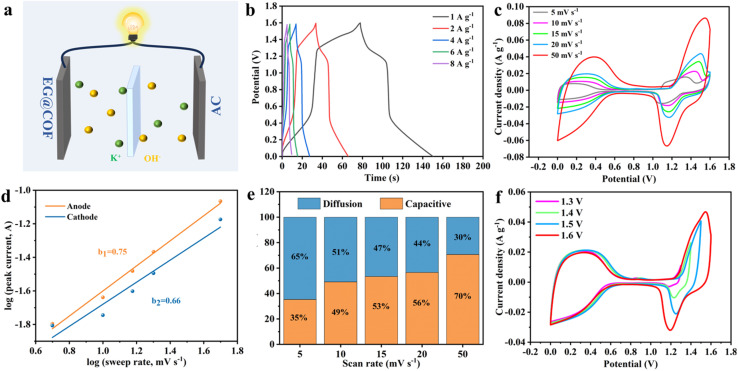
(a) Schematic of the AC//EG@COF device, (b) GCD plots, (c) CV plots, (d) *b*-values, (e) capacitive and diffusion contribution, (f) CV plots for different voltage windows.

To further investigate the electrochemical behavior of EG@COF, an asymmetric supercapacitor (AC//EG@COF-3) was prepared following the method by Zhu^30^ (Fig. 5a). The GCD curve of the ACS is shown in Fig. 5b. The specific capacity of the ACS was calculated to be 72 C g^−1^ at 1 A g^−1^. Its CV curves at different scanning rates are summarized in Fig. 5c. The non-rectangular shape of the curves indicated the simultaneous presence of pseudo-capacitance and double-layer capacitance. Additionally, the relationship between the peak current (*i*) and scanning rate (*v*) can be described by eqn (5):

5 *i* = *av*^*b*^

6 *i*(*V*) = *k*_1_*v* + *k*_2_*v*^1/2^,

where *i* is the current (A), *v* is the scan rate (V s^−1^), *a* and *b* are variable parameters, and *k*_1_ and *k*_2_ are constants.

Usually, the *b*-value of 0.5 indicates linear diffusion control, while a *b*-value of 1.0 indicates capacitive charge storage without diffusion control. As shown in Fig. 5d, the *b*-values were calculated to be 0.75 and 0.66 based on the oxidation and reduction peaks of EG@COF. This implied that the electrochemical behavior of EG@COF was both capacitive and diffusion-controlled. The ratio of capacitive and diffusive contributions was calculated by eqn (6). The proportions of capacitive and diffusive contributions for scan rates of 5–50 mV s^−1^ are shown in Fig. 5e. The capacitive contribution values were found 35%, 49%, 53%, 56% and 70%, respectively. The result illustrated that the capacitive contribution gradually increased and dominated as the scan rate increased. Fig. 5f summarizes the CV curves of the AC//EG@COF device with different voltages at a scan rate of 20 mV s^−1^. No significant polarisation was observed even at 1.6 V, verifying the feasibility of the voltage range selection.

**Fig. 6:**
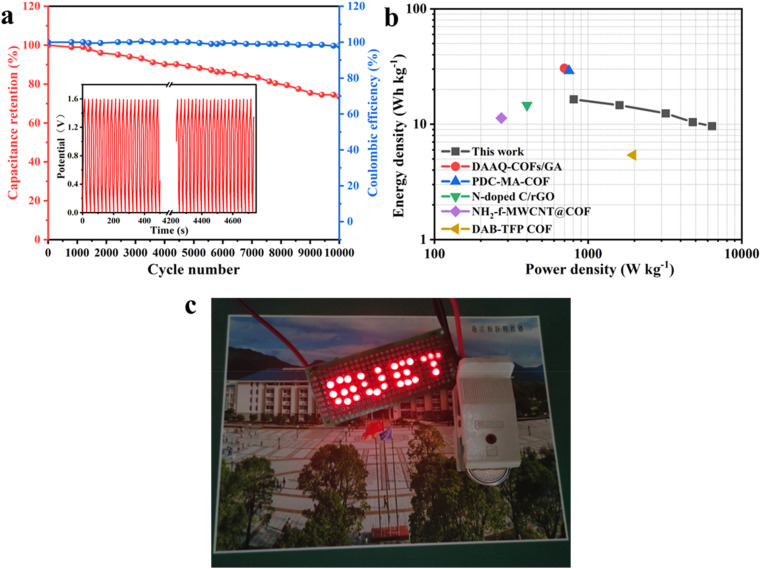
(a) Cycling stability test, (b) Ragone plot of the ASC, (c) digital photograph of LEDs powered by ASC.

In practical applications, energy density and power density are two indicators used to evaluate the performance of materials. As shown in Fig. 6b, the energy density of EG@COF//AC was 16.4 W h kg^−1^ at a power density of 806.0 W kg^−1^, exceeding the electrode materials reported in ref. 19 and 31. Fig. 6a shows that the capacitance retention of EG@COF//AC reached 74% after 10 000 cycles at 4 A g^−1^, while the coulombic efficiency remained at 98%. The above results proved that EG@COF had good cycling stability. In addition, it could also be observed from the inset that the GCD curves before and after cycling remained stable. Fig. 6c demonstrated that a GUET panel consisting of 29 LEDs could be lit using two EG@COF//AC devices connected in series.

## 4. Conclusion

Layered porous material EG@COF was successfully synthesized by a solvent-free *in situ* synthesis method. Here, EG was used as the growth substrate of COF, enabling effective charge transport within EG (as a conductive skeleton), thereby improving the conductivity of the COF and modulating the pore size of COF to a certain extent. Therefore, more redox active sites could be utilized. The ion exchange rate was accelerated by the synergistic effect of EG and COF. Electrochemical tests demonstrated that the specific capacitance of EG@COF-3 was as high as 351 C g^−1^ at 1 A g^−1^. The capacitance retention was 94.4% after 10 000 cycles. It exhibited good performance when used as an EG@COF//AC device. The ongoing optimization of COFs focuses not only on the adjustment of pore size but also on the development of COF species and the utilization of redox sites. This enhancement of COF conductivity and regulation of the pore size of COF through a carbon growth substrate, as demonstrated in this study, also provides new ideas for the application of COF-based composites in the field of energy storage.

## Data availability

The data will be made available upon request.

## Conflicts of interest

The authors declare that there is no conflict of interest regarding the publication of this paper.

## Supplementary Material

RA-015-D5RA01653C-s001
