# Supplementary material for: Enhanced supercapacitor performance using EG@COF: a layered porous composite
Source: RSC Adv. 2025 Apr 11;15(15):11441–50. doi: 10.1039/d5ra01653c (PMC11987848; doi:10.1039/d5ra01653c)
Supplement: RA-015-D5RA01653C-s001 [file RA-015-D5RA01653C-s001.pdf]

Supplementary Information (SI) for RSC Advances.
This journal is © The Royal Society of Chemistry 2025

Enhanced Supercapacitor Performance Using EG@COF: A Novel Layered Porous Composite

Junaid Khan[a, b, *], Anique Ahmed[c, d], Abdullah A. Al-Kahtani[e]

[a]Department of Physics, Government Postgraduate Collage No.1, Abbottabad, Khyber Pakhtunkhwa, Pakistan
[b]Department Of Higher Education Achieves and Libraries, Government of Khyber Pakhtunkhwa, Pakistan
[c]Faculty of Engineering Sciences, GIK Institute of Engineering Sciences and Technology, Topi 23640, Khyber Pakhtunkhwa, Pakistan
[d]Department of Chemical and Bilogical Engineering, Gachon University, 1342 Seongnam-daero, Seongnam13120, Republic of Korea
[e]Chemistry Department, Collage of Science, King Saud University, P. O. Box 2455, Riyadh-22451, Saudi Arabia

*Email: junaidkhan.nanotech@gmail.com

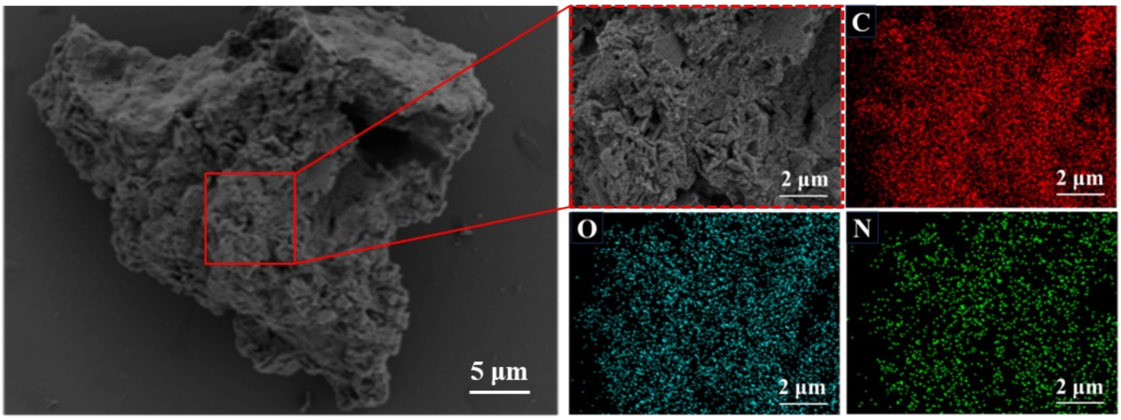

Fig. S1. EDS diagram of EG@COF-3

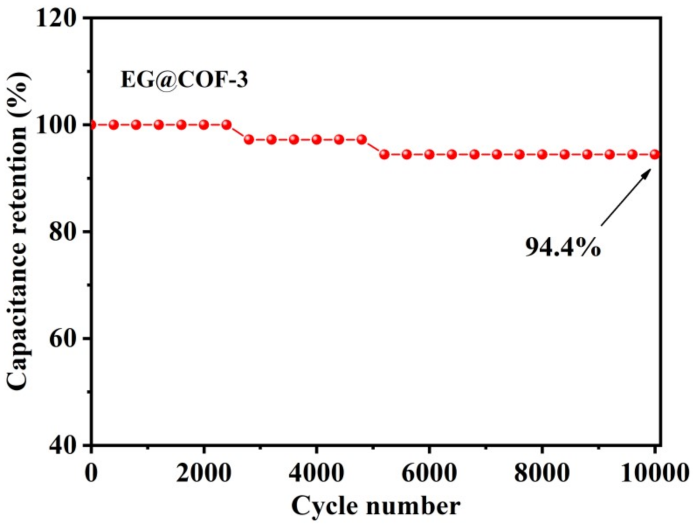

Fig. S2. Cycling stability of EG@COF-3 measured at 10 A g^{-1} for 10 000 cycles.
